# Prognostic Indicators of Morbidity and Mortality in Children with Congestive Hepatopathy Presenting with Ascites

**DOI:** 10.3390/diagnostics14151618

**Published:** 2024-07-26

**Authors:** Harisa Spahic, Paul Wasuwanich, Bahareh Modanloo, Songyos Rajborirug, Shelby Kutty, Ari Cedars, Wikrom Karnsakul

**Affiliations:** 1 Department of Pediatrics, University of Colorado, Aurora, CO 80045, USA; 2 Department of Internal Medicine, University of Florida College of Medicine, Gainesville, FL 32610, USA; 3 Biostatistics, Epidemiology, and Data Management (BEAD) Core, Department of Pediatrics, Johns Hopkins University School of Medicine, Baltimore, MD 21205, USA; 4 Department of Epidemiology, Johns Hopkins University Bloomberg School of Public Health, Baltimore, MD 21205, USA; 5 Division of Pediatric Cardiology, Department of Pediatrics, Johns Hopkins University School of Medicine, Baltimore, MD 21205, USA; 6 Division of Pediatric Gastroenterology, Hepatology, and Nutrition, Department of Pediatrics, Johns Hopkins University School of Medicine, 550 N. Broadway, 10th Floor Suite 1003, Baltimore, MD 21205, USA

**Keywords:** pediatrics, congestive hepatopathy, right-sided heart disease, chronic liver disease, portal hypertension

## Abstract

***Objectives:*** Congestive hepatopathy is a significant complication for children suffering from right-sided heart disease (RHD). We hypothesize that hospitalized pediatric patients with ascites will have congestive hepatopathy leading to advanced liver disease if their cardiac condition is RHD versus non-right-sided heart disease (NRHD). ***Methods:*** This is a retrospective cohort study of pediatric patients who presented with an ascites diagnosis (ICD-10 R18) and at least one cardiac diagnosis. Patient demographics, past medical history, laboratory values, imaging results, calculated clinical scores (e.g., APRI, FIB-4), treatment, length of stay (LOS), and death at hospital discharge were analyzed. ***Results:*** Of the 136 patients with ascites, 21 patients presented with a primary cardiac disease (12 in RHD and 9 in NRHD). Of these patients, eight (38%) were female, and nine (43%) were White, seven (33%) were Black, and five (24%) were unknown. The RHD group had a mean age of 5.1 Y (vs. 9.5 Y in NRHD). The mean APRI score in RHD patients was 2.87, and it was 0.85 in NRDH. Treatments were similar, with most patients requiring diuretics (11 RHD (92%) vs. 8 NRDH (89%)); 5 RHD (42%) vs. 4 NRDH (44%) required inotropic support. RHD patients had a longer LOS, with an average of 92 days vs. 52 days for NRDH patients. Overall, each group had one death at discharge (8% RHD vs. 11% NRDH). ***Conclusions:*** In the realm of children with ascites, the subset grappling with congestive heart disease paints a unique picture. In this context, ascites stands as an elusive predictor of liver decompensation, defying conventional diagnostic pathways.

## 1. Introduction

The heart–liver axis refers to the bidirectional effects of these two organs on one another, especially when the pathophysiology of one impacts the other [1,2,3,4]. Specifically, liver disease that results from heart disease has been generally referred to as “cardiac hepatopathy”, although consensus on the terminology is lacking. There are two main forms of cardiac hepatopathy, acute cardiogenic liver injury (also referred to as hypoxic hepatitis) and congestive hepatopathy [5,6]. Congestive hepatopathy is characterized by the congestion of the liver parenchyma induced by impaired hepatic venous outflow secondary to cardiac dysfunction [7,8,9,10,11,12,13]. While occurring at all ages, pediatric congestive hepatopathy is unique, as it tends to affect patients with congenital cardiac lesions signifying that the liver has almost always experienced a congestive state [3,14,15,16].

The pathophysiology of congestive hepatopathy is complex but increasingly understood; most researches within the pediatric population have been performed with those who underwent a Fontan procedure, which is on the rise as well [2,15]. The liver is a highly vascular organ with a dual blood supply, receiving up to 25% of the total cardiac output, but it is highly vulnerable to congestion. When congestion occurs, it damages the liver through four main pathogenic mechanisms: (1) shear stress contributes to fibrogenesis and sinusoidal ischemia; (2) decreased portal vein inflow due to a reduced hepatic venous pressure gradient worsens hepatic ischemia; (3) the accumulation of exudate further encourages fibrogenesis; and (4) sinusoidal thrombosis contributes to liver fibrosis [7,8,9,10,11]. Specifically, inflammation is not suspected to contribute to the fibrosis observed [11]. Ultimately, any cause of right-sided heart disease, such as tricuspid regurgitation, mitral stenosis, severe constrictive pericarditis, or end-stage cardiomyopathies, can lead to congestive hepatopathy. Consequentially, pediatric patients with congestive hepatopathy are a rare group and are increasingly more difficult to study, given the small size of the affected individuals.

In general, pediatric patients with cardiac disease that eventually contributes to congestive hepatopathy are typically asymptomatic with respect to their liver disease. Rarely, those with hepatic congestion may present with mild jaundice, ascites, or right upper quadrant pain, due to the expansion of the liver. Among those symptoms, ascites is a frequent finding, but it does not necessary mean that liver cirrhosis has already developed [12]. Nevertheless, the symptoms of cardiac failure typically dominate the clinical presentation. The presence of cardiac dysfunction with the potential to cause elevated central venous pressure is a common indication for the clinical evaluation of passive congestion [13]. Crucially, it is possible that many patients who are affected may not be recognized and diagnosed with congestive hepatopathy, thereby increasing the rarity of finding patients to include in studies.

Screening for congestive hepatopathy in children with cardiac dysfunction remains a challenge, as current methods yield inconsistent results [15,17,18,19]. A common problem seen in patients hospitalized with congestive hepatopathy is ascites, due to the high pressures in the gastrointestinal vasculature, concurrent with low osmotic pressure and high volume states [3]. Compared to other pediatric patients with ascites, those with congestive hepatopathy have higher rates of morbidity and mortality [20,21,22]. This finding highlights the need to better understand this disease pathophysiology and potentially screen for earlier detection and treatment [20]. However, it is unclear what influences the patient’s morbidity and mortality when the modulating factors contributing to the development and severity of hepatic fibrosis remain unknown [23,24,25]. Further clouding the picture is the development of ascites, which can result from cardiac or hepatic dysfunction, whereby determining the etiology of ascites is difficult [26].

We hypothesize that pediatric patients who are found to have ascites during hospitalizations will have different clinical variables based on whether their cardiac dysfunction is right-sided heart disease (RHD) versus non-right-sided heart disease (NRHD). Furthermore, we hypothesize that those with RHD will have pathophysiology promoting congestive hepatopathy, therefore being more likely to demonstrate worse liver dysfunction than those with NRHD.

## 2. Methods

### 2.1. Study Design and Population

**Figure 1:**
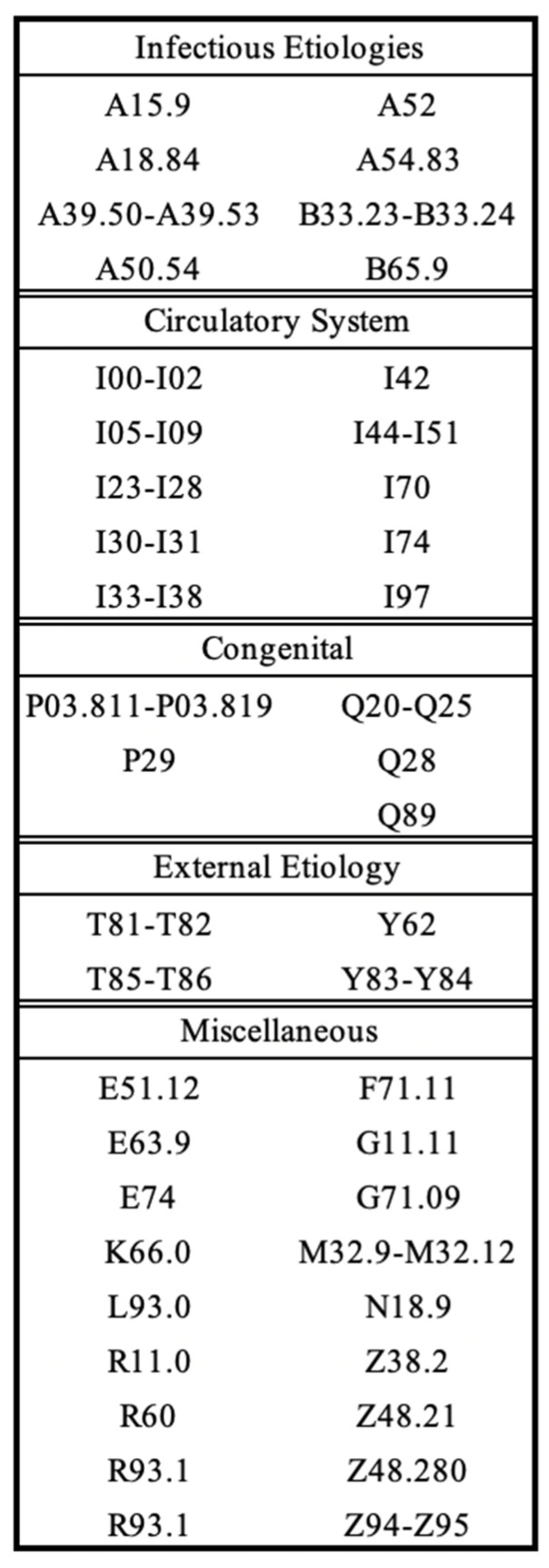
International Classification of Diseases, Tenth Revision (ICD-10) diagnostic codes for cardiac etiologies.

**Figure 2:**
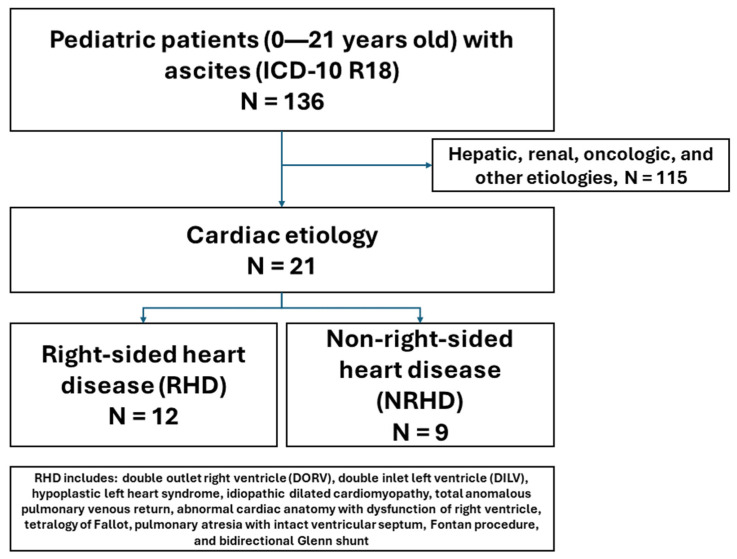
Schematic for the selection of the study population. All children admitted to John’s Hopkins Children Center with ascites were first identified via the ICD-10 code for ascites. Charts were reviewed to determine the etiology of ascites, whereby only those with a cardiac etiology of ascites were included. Excluded patients (total *N* = 115) had ascites due to a hepatic etiology (*N* = 34), oncologic etiology (*N* = 24), renal etiology (*N* = 12), or other/complex etiology (*N* = 45). Those with right-sided heart disease (RHD) included the following conditions: double outlet right ventricle (DORV), double inlet left ventricle (DILV), hypoplastic left heart syndrome, idiopathic dilated cardiomyopathy, total anomalous pulmonary venous return, abnormal cardiac anatomy with dysfunction of the right ventricle, tetralogy of Fallot, pulmonary atresia with intact ventricular septum, Fontan procedure, and bidirectional Glenn shunt.

This study was a retrospective cohort study of patients aged 0 to 21 years old with a primary cardiac disease admitted to the Children’s Center at Johns Hopkins Hospital in Baltimore, MD from 1 January 2016 to 31 December 2022 with new onset ascites. Identification of patients was performed in collaboration with Johns Hopkins BEAD Core via automated search through electronic medical records utilizing the International Classification of Diseases, Tenth Revision (ICD-10) diagnostic codes. Ascites was defined using the ICD-10 code of R18.0 or R18.8 for the diagnosis of all types of ascites at hospital discharge. Cardiac conditions were identified by ICD-10 codes that corresponded to the diagnoses of cardiac pathology described in the following subsection (Figure 1). These diagnoses can occur during any encounter prior to the presence of ascites. Any subsequent hospitalizations with a diagnosis of ascites for the same patient were excluded in this study. Patients with other primary disease processes, such as oncological or primary liver disease, were excluded (Figure 2). This study (number IRB00275901) was approved by the Institutional Review Board of the Johns Hopkins University School of Medicine.

### 2.2. Cardiac Pathology Classifications

Cardiac dysfunction was classified into two groups: right-sided heart disease (RHD) and other cardiac conditions, which we referred to as non-right-sided heart disease (NRHD). Patients in the RHD group had at least one of the following problems in their past medical history: total anomalous pulmonary venous return (TAPVR) with pulmonary hypertension, hypoplastic left heart syndrome (HLHS), tetralogy of Fallot (ToF), double outlet right ventricle (DORV), double inlet left ventricle (DILV), abnormal cardiac anatomy necessitating a Fontan procedure, and/or dysfunction of the right ventricle (Figure 1).

### 2.3. Study Variables

Demographic and clinical data for the first admission with ascites were collected by data scientists at the BEAD Core at the Johns Hopkins University. The data collected included patient demographics (e.g., age and race), past medical history (e.g., esophageal varices, splenomegaly, and hepatic encephalopathy), laboratory values (e.g., platelets), imaging results (e.g., echocardiogram ejection fraction [EF]), treatment (e.g., diuretic use and inotrope use), length of hospital stay (LOS), and death at hospital discharge. Based on these data, clinical scores, including the aspartate aminotransferase to platelet ratio index (APRI) and fibrosis-4 (FIB-4), were calculated. All data were then compared between the two groups. Of note, the APRI score was calculated by the following formula:

$$\frac{\frac{\mathrm{Patient}\;\mathrm{AST}\;\mathrm{Value}\;(U/L)}{\mathrm{Upper}\;\mathrm{Limit}\;\mathrm{of}\;\mathrm{Normal}\;\mathrm{for}\;\mathrm{AST}\;(40\;U/L)}}{\mathrm{Patient}\;\mathrm{Platelet}\;\mathrm{Count}\;(10^{9}/L)}$$

The FIB-4 score was calculated by the following formula:

$$\frac{\mathrm{Patient}\prime s\;\mathrm{Age}\;(\mathrm{Years})\times \mathrm{Patient}\prime s\;\mathrm{AST}\;\mathrm{Value}\;(U/L)}{\mathrm{Patient}\prime s\;\mathrm{Platelet}\;\mathrm{Count}\;(10^{9}/L)\times \surd \mathrm{Patient}\prime s\;\mathrm{ALT}\;\mathrm{Value}\;(U/L)}$$

### 2.4. Statistical Analysis

The statistical analysis was completed by utilizing all data available. Given the small sample size between the two groups, Student’s *t*-test and Fischer’s exact test were used to assess the differences between the groups. Statistical significance was defined as having a *p*-value of less than 0.05.

## 3. Results

### 3.1. Population Demographics

We identified a total of 136 pediatric patients with ascites, among whom there were 21 (15.4%) patients who presented with a primary cardiac disease with ascites (12 in RHD and 9 in NRHD). Of these patients with primary cardiac disease, eight (38%) were female. The race/ethnic distribution was nine (43%) White, seven (33%) Black, and five (24%) unknown. Those in the RHD group had a mean age of 5.1 years, compared to 9.5 years in the NRHD group.

### 3.2. Clinical Features

With regards to physical exam findings, hepatomegaly was observed in seven (58%) RHD patients, compared to five (56%) in the NRHD group, while splenomegaly was observed in six RHD patients (50%) and only three NRDH patients (33%). Interestingly, none of the patients had a history of esophageal varices or hepatic encephalopathy upon review of their past medical histories (Table 1).

### 3.3. Laboratory Results

Upon review of the lab results, those with RHD tended to have elevated transaminases, while those with NRHD tended to have lower platelet values. RHD patients had an average AST of 145.9 U/L and alanine aminotransferase (ALT) of 61.5 U/L, compared to NRHD, with an average AST of 34.9 U/L and an average ALT of 24.6 U/L. Mean platelet values for RHD patients was 216.7 × 10^9^/L, and for NRHD patients, it was 140.0 × 10^9^/L (Table 1).

Utilizing known formulas to assess the degree of liver dysfunction and degrees of portal hypertension, the mean APRI score in RHD patients was 2.87, and in NRHD patients, it was 0.85. The mean FIB-4 scores were 0.66 and 0.46 in RDH patients and NRDH patients, respectively (Table 1).

### 3.4. Imaging Findings

When echocardiography imaging results were reviewed, patients with RHD had a mean right atrial pressure of 9.65 mmHg, while those with NRDH had a mean right atrial pressure of 9.17 mmHg. On the left side of the heart, those with RHD had an average left ventricular ejection fraction of 54.4%, while those with NRHD had an average of 48.3%. However, half of the NRDH patients had no right atrial pressure assessment, and almost half of the RHD patients did not have a left ventricular ejection fraction value on their echocardiography report (Table 1). This difference was typically related to abnormal cardiac anatomy, which prevented the measurement of a right atrial pressure or ejection fraction, since a right atrium or left ventricle was not present to provide an opportunity for that measurement.

### 3.5. Clinical Treatments and Outcomes

With regards to treatments, both groups had similar treatment regimens. Most patients required diuretics, with 11 (92%) patients in the RHD group versus 8 (89%) patients in the NRDH group. Fewer patients required inotropic support, with five (42%) patients in the RHD group compared to four (44%) patients in the NRDH group (Table 1).

Patients with RHD had a longer LOS, with an average of 92 days versus 52 days for patients with NRDH. Overall, each group had one death at discharge (8% RHD vs. 11% NRDH) (Table 1).

No statistically significant results were found, given the small group sizes.

## 4. Discussion

Although ascites is a clinical hallmark in and predictor of advanced liver disease with portal hypertension, there are limited data for children with a primary heart disease who present to the hospital with ascites. Importantly, this study builds upon previous research, which found that children with congestive hepatopathy as the cause for their ascites had higher morbidity and mortality among pediatric patients with ascites [20,21]. To better understand and differentiate this group, this study sought to understand the clinical and laboratory differences between two types of heart disease. We hypothesized that patients with right-sided heart dysfunction (and hence a higher likelihood of congestive hepatopathy) would have distinct clinical findings, compared to those without right-sided heart dysfunction, and we indeed found multiple variables that trended differently between these two populations.

APRI and FIB-4 scores, which have historically been used to assess liver dysfunction or portal hypertension [15,27,28,29,30] and have more recently been recommended as first-line testing to assess for portal hypertension [31], were notably different between the two groups. In this study, we found that those with RHD had an average APRI of 2.87 ± 2.62, while those with NRHD had an APRI of 0.85 ± 0.8, suggesting portal hypertension in most patients with RHD. However, there was a smaller magnitude of difference within the FIB-4 score (0.66 ± 0.98 in RHD vs. 0.46 ± 0.69 in NRHD), and neither group, on average, reached the threshold of >3.25 typically used for portal hypertension from cirrhosis [30]. This difference is interesting, as both scores utilized AST and platelet values in their calculation, but the FIB-4 score also incorporated age and ALT. Of note, the mean age in RHD was lower than in NRHD, while the mean ALT was higher. Despite the APRI and FIB-4 scores lacking specific relation to liver fibrosis in cases with congestive hepatopathy [7], the difference remains challenging, as relatively high APRI and FIB-4 scores may indicate an early course of liver disease in congestive hepatopathy from hepatic congestion rather than from advanced fibrosis.

For the age component, an inherent bias existed in the calculation for lower scores for younger patients, despite previous literature finding that many children with congenital heart diseases, especially those with Fontan procedures who are at high risk for liver dysfunction, tend to be younger [15,32,33,34,35,36]. Furthermore, this result of younger patients in the RHD group is similar to previous work that found that those with congestive hepatopathy tended to be younger than those with other causes for ascites [21]. It is possible that the age of the RHD group was influenced by the presence of seven patients with a history of the Fontan procedure who were known to be younger than those who had other types of RHD [16].

Additionally, the differences between AST and ALT were also stark; patients with RHD had higher values of AST, 145.9 ± 135 U/L and ALT 61.5 ± 94.4 U/L, compared to patients with NRHD (AST 34.9 ± 27.3 U/L and ALT 24.6 ± 15.4 U/L). This result is indicative of those with RHD undergoing an acute process affecting their liver, while those with NRHD did not showcase the typical AST/ALT rise seen with an acute insult to the liver [1,37]. The elevated AST and ALT values in patients with RHD corresponded to previous literature, where cardiac patients with the progression of liver disease, especially early in the course, tended to have higher AST and ALT values [2,4,27].

The other two components of portal hypertension are the presence of varices and splenomegaly. Previous literature have found that patients with liver disease developed in the setting of cardiac disease were less likely to have ascites [29,33,38]. Conversely, splenomegaly was seen in both groups but more commonly in patients with RHD (50%), compared to 33% in the NRHD group.

Unsurprisingly, there was not a substantial difference in the proportion of diuretic use as the treatment between the two groups. Almost all the patients were treated with a diuretic, which reduced overall fluid status, thereby helping to reduce a patient’s central venous/right atrial/right ventricular pressure.

One of the most substantial differences found between the two groups was that those with RHD had, on average, longer lengths of stay in the hospital, compared to those with NRHD (92.2 ± 146 days vs. 51.9 ± 71.5 days, respectively). The underlying reason for the length of stay was not able to be determined within the scope of this study, but it can provide prognostic value to the clinician treating the patient with ascites during their admission based on the patient’s cardiac disease. Limited literatures exist as guidance for the clinician when treating a cardiac patient with ascites. Most of the research performed are specifically for patients with a history of the Fontan procedure, with a mixture of adult and pediatric populations [18,32,39,40,41].

While the resultant liver dysfunction from cardiac dysfunction is challenging to treat, new trials have shown that a heart transplantation for those with Fontan-associated liver disease improves their markers of liver dysfunction, and long-term outcomes are favorable [35,38,42,43,44]. Even if liver dysfunction is severe, promising results have also been seen with en bloc heart–liver transplants [45,46].

Overall, the presence of cardiac disease was relatively low among children with ascites in this retrospective 7-year study (21 cardiac patients out of 136 total patients). This is consistent with other studies of pediatric ascites, which have also found a relatively low prevalence of congestive hepatopathy in their cohorts [20,21,47]. Consequentially, it is crucial to assess trends in data, given the small sample sizes for rarer conditions. Ascites is generally uncommon in children, with most etiologies being related to liver pathology [3]. Among our small group of pediatric patients with primary cardiac pathology presenting with ascites, an overall trend emerged, differentiating those with a right-sided heart disease and those with a non-right-sided heart disease. Children in the RHD group appeared to have more evidence of portal hypertension than the NRHD group. Furthermore, those with RHD tended to have higher morbidity. However, the treatment and incidence of death between these two groups did not differ notably. A further longitudinal study with a larger sample size and inclusion of children with cardiac disease without ascites should focus on exploring the association between the presence of ascites and the subsequent occurrence of portal hypertension among pediatric patients with cardiac disease.

In children with a known cardiac pathology, screening for or investigating for congestive hepatopathy should be considered. Although only 15.4% of pediatric ascites patients in our cohort had a cardiac etiology for the ascites, congestive hepatopathy should be on the differential diagnosis when presented with a pediatric patient with ascites. The diagnosis of congestive hepatopathy is multifactorial, relying on a combination of multiple findings from clinical presentation, laboratory results, and imaging; there is currently no widely accepted algorithm for diagnosis.

## 5. Limitations

The study’s retrospective nature and single-center design, coupled with a modest patient cohort, constrained the capacity to ascertain statistical significance and infer causality among variables. The rarity of this condition in a pediatric population contributes to this limitation. However, the rarity also highlights the importance of continuing to study this condition, even in small cohorts, to better start to understand any differences in the groups and begin discussions to help this cohort of patients. Moreover, the inclusion of solely ascitic pediatric patients restricts comparisons with those without, potentially skewing toward a cohort with lower incidences or milder forms of congestive hepatopathy. Future investigations can leverage these preliminary findings to delineate specific variables for collection and analysis across a larger and more diverse patient population. For conditions which are rare, such as congestive hepatopathy in pediatric patients, it would be favorable to conduct multi-center studies to increase the potential study size.

Future studies could also focus on all patients with a primary cardiac etiology, then compare risk factors between those who were found to have ascites versus no ascites. This would not only increase the size of the study population but allow for the better determination of group characteristics between those with RHD and NRHD. Given the above results, it is possible that many patients were not included, given that ascites can sometimes not be noted during admission.

## 6. Conclusions

Among pediatric patients presenting with ascites, cardiac disease is infrequently encountered. Conversely, providers treating pediatric patients with cardiac disease do not have validated or readily available tools to monitor for the progression of liver disease that could include ascites. Alternative markers such as APRI and FIB-4 scores or liver imaging could serve as more reliable indicators of liver fibrosis or portal hypertension. Notably, children afflicted with both ascites and RHD exhibited notably higher morbidity rates compared to their NRHD counterparts. However, mortality outcomes remained similar across both groups. These findings underscore the necessity for further investigation with a broader study cohort to elucidate these associations conclusively.

## Figures and Tables

**Table 1:** Clinical characteristics and laboratory findings of patients with ascites and right-sided heart disease (RHD) and non-right-side heart disease (NRHD).

		RHD (N = 12)	NRHD (N = 9)
**History of**			
Esophageal Varices	N (%)	0 (0.0)	0 (0.0)
Splenomegaly	N (%)	6 (50.0)	3 (33.3)
Hepatomegaly	N (%)	7 (58.3)	5 (55.6)
Encephalopathy	N (%)	0 (0.0)	0 (0.0)
Jaundice	N (%)	2 (16.7)	1 (11.1)
**Lab/Imaging Results**			
Platelets (10^9/L)	Mean (SD)	216.7 (18.1)	140.0 (86.3)
AST (U/L)	Mean (SD)	145.9 (135.0)	34.9 (27.3)
ALT (U/L)	Mean (SD)	61.5 (94.4)	24.6 (15.4)
RA Pressures (mmHg)	Mean (SD)	9.65 (5.2)	9.17 (6.0)
LV EF (%)	Mean (SD)	54.4 (18.8)	48.3 (20.2)
**Calculated Scores**			
APRI	Mean (SD)	2.87 (2.62)	0.85 (0.80)
FIB-4	Mean (SD)	0.66 (0.98)	0.46 (0.69)
**Treatment**			
Diuretics	N (%)	11 (91.7)	8 (88.9)
Inotropes	N (%)	5 (41.7)	4 (44.4)
**Outcome**			
LOS (days)	Mean (SD)	92.2 (146.0)	51.9 (71.5)
Death at Discharge	N (%)	1 (8.3)	1 (11.1)

## Data Availability

The data presented in this study are available on request from the corresponding author. The data are not publicly available due to restrictions e.g., privacy or ethics.

## References

[1] El Hadi H., Di Vincenzo A., Vettor R., Rossato M. (2020). Relationship between Heart Disease and Liver Disease: A Two-Way Street. Cells.

[2] Fortea J.I., Puente Á., Cuadrado A., Huelin P., Pellón R., González Sánchez F.J., Mayorga M., Cagigal M.L., García Carrera I., Cobreros M. (2020). Congestive Hepatopathy. Int. J. Mol. Sci..

[3] Giefer M.J., Murray K.F., Colletti R.B. (2011). Pathophysiology, diagnosis, and management of pediatric ascites. J. Pediatr. Gastroenterol. Nutr..

[4] Naschitz J.E., Slobodin G., Lewis R.J., Zuckerman E., Yeshurun D. (2000). Heart diseases affecting the liver and liver diseases affecting the heart. Am. Heart J..

[5] Ford R.M., Book W., Spivey J.R. (2015). Liver disease related to the heart. Transplant. Rev..

[6] Henrion J. (2012). Hypoxic hepatitis. Liver Int..

[7] Lemmer A., VanWagner L.B., Ganger D. (2018). Assessment of Advanced Liver Fibrosis and the Risk for Hepatic Decompensation in Patients with Congestive Hepatopathy. Hepatology.

[8] Hilscher M., Sanchez W. (2016). Congestive hepatopathy. Clin. Liver Dis..

[9] Marra F., DeFranco R., Grappone C., Milani S., Pinzani M., Pellegrini G., Laffi G., Gentilini P. (1998). Expression of the thrombin receptor in human liver: Up-regulation during acute and chronic injury. Hepatology.

[10] Wanless I.R., Liu J.J., Butany J. (1995). Role of thrombosis in the pathogenesis of congestive hepatic fibrosis (cardiac cirrhosis). Hepatology.

[11] Kiesewetter C.H., Sheron N., Vettukattill J.J., Hacking N., Stedman B., Millward-Sadler H., Haw M., Cope R., Salmon A.P., Sivaprakasam M.C. (2007). Hepatic changes in the failing Fontan circulation. Heart.

[12] Myers R.P., Cerini R., Sayegh R., Moreau R., Degott C., Lebrec D., Lee S.S. (2003). Cardiac hepatopathy: Clinical, hemodynamic, and histologic characteristics and correlations. Hepatology.

[13] Giallourakis C.C., Rosenberg P.M., Friedman L.S. (2002). The liver in heart failure. Clin. Liver Dis..

[14] Elder R.W., McCabe N.M., Hebson C., Veledar E., Romero R., Ford R.M., Mahle W.T., Kogon B.E., Sahu A., Jokhadar M. (2013). Features of portal hypertension are associated with major adverse events in Fontan patients: The VAST study. Int. J. Cardiol..

[15] Komatsu H., Inui A., Kishiki K., Kawai H., Yoshio S., Osawa Y. (2019). Liver disease secondary to congenital heart disease in children. Expert Rev. Gastroenterol. Hepatol..

[16] Yoo B.W., Choi J.Y., Eun L.Y., Park H.K., Park Y.H., Kim S.U. (2014). Congestive hepatopathy after Fontan operation and related factors assessed by transient elastography. J. Thorac. Cardiovasc. Surg..

[17] Dillman J.R., Trout A.T., Alsaied T., Gupta A., Lubert A.M. (2020). Imaging of Fontan-associated liver disease. Pediatr. Radiol..

[18] Rathgeber S.L., Guttman O.R., Lee A.F., Voss C., Hemphill N.M., Schreiber R.A., Harris K.C. (2020). Fontan-Associated Liver Disease: Spectrum of Disease in Children and Adolescents. J. Am. Heart Assoc..

[19] Rathgeber S.L., Harris K.C. (2019). Fontan-Associated Liver Disease: Evidence for Early Surveillance of Liver Health in Pediatric Fontan Patients. Can. J. Cardiol..

[20] Ingviya T., Wasuwanich P., Scheimann A.O., Felix G., Laengvejkal P., Vasilescu A., Imteyaz H., Seaberg E.C., Karnsakul W. (2021). Clinical Predictors of Morbidity and Mortality in Hospitalized Pediatric Patients with Ascites. J. Pediatr. Gastroenterol. Nutr..

[21] Karnsakul W., Ingviya T., Seaberg E., Laengvejkal P., Imteyaz H., Vasilescu A., Schwarz K.B., Scheimann A.O. (2017). Ascites in Children: A Single-Center Experience of 27 Years. J. Pediatr. Gastroenterol. Nutr..

[22] Dai D.F., Swanson P.E., Krieger E.V., Liou I.W., Carithers R.L., Yeh M.M. (2014). Congestive hepatic fibrosis score: A novel histologic assessment of clinical severity. Mod. Pathol..

[23] Dori Y., Mazurek J., Birati E., Smith C. (2023). Ascites in Animals with Right Heart Failure: Correlation with Lymphatic Dysfunction. J. Am. Heart Assoc..

[24] Hidaka H., Iwakiri Y. (2015). Hepatic congestion leads to fibrosis: Findings in a newly developed murine model. Hepatology.

[25] Simonetto D.A., Yang H.-Y., Yin M., de Assuncao T.M., Kwon J.H., Hilscher M., Pan S., Yang L., Bi Y., Beyder A. (2015). Chronic passive venous congestion drives hepatic fibrogenesis via sinusoidal thrombosis and mechanical forces. Hepatology.

[26] Lane E.R., Hsu E.K., Murray K.F. (2015). Management of ascites in children. Expert Rev. Gastroenterol. Hepatol..

[27] De Lange C., Möller T., Hebelka H. (2023). Fontan-associated liver disease: Diagnosis, surveillance, and management. Front. Pediatr..

[28] Emmalee J., Khan S., Weaver C., Goldbeck C., Yanni G., Kohli R., Genyk Y., Zhou S., Shillingford N., Sullivan P.M. (2021). Non-invasive biomarkers of Fontan-associated liver disease. JHEP Rep. Innov. Hepatol..

[29] Hilscher M.B., Wells M.L., Venkatesh S.K., Cetta F., Kamath P.S. (2022). Fontan-associated liver disease. Hepatology.

[30] Hilscher M.B., Johnson J.N., Cetta F., Driscoll D.J., Poterucha J.J., Sanchez W., Connolly H.M., Kamath P.S. (2017). Surveillance for liver complications after the Fontan procedure. Congenit. Heart Dis..

[31] Duarte-Rojo A., Patel K., Rockey D.C. (2024). Non-invasive assessment of liver fibrosis and portal hypertension. Curr. Opin. Gastroenterol..

[32] De Bruyne R., Vandekerckhove K., Van Overschelde H., Hendricx F., Vande Walle C., De Groote K., Panzer J., De Wolf D., Van Biervliet S., Bové T. (2022). Non-invasive assessment of liver abnormalities in pediatric Fontan patients. Eur. J. Pediatr..

[33] Emamaullee J., Zaidi A.N., Schiano T., Kahn J., Valentino P.L., Hofer R.E., Taner T., Wald J.W., Olthoff K.M., Bucuvalas J. (2020). Fontan-Associated Liver Disease: Screening, Management, and Transplant Considerations. Circulation.

[34] Gordon-Walker T.T., Bove K., Veldtman G. (2019). Fontan-associated liver disease: A review. J. Cardiol..

[35] Griffiths E.R., Lambert L.M., Ou Z., Shaaban A., Rezvani M., Carlo W.F., Schumacher K.R., DiPaola F., O’Connor M.J., Nandi D. (2023). Fontan-associated liver disease after heart transplant. Pediatr. Transplant..

[36] Inuzuka R., Nii M., Inai K., Shimada E., Shinohara T., Kogiso T., Ono H., Otsuki S., Kurita Y., Takeda A. (2023). Predictors of liver cirrhosis and hepatocellular carcinoma among perioperative survivors of the Fontan operation. Heart.

[37] Koehne de Gonzalez A.K., Lefkowitch J.H. (2017). Heart Disease and the Liver: Pathologic Evaluation. Gastroenterol. Clin. N. Am..

[38] Broda C.R., Alonso-Gonzalez R., Ghanekar A., Gulamhusein A., McDonald M., Luk A., Kobulnik J., Billia F., Heggie J., Jariani M. (2022). Fate of the liver in the survivors of adult heart transplant for a failing Fontan circulation. J. Heart Lung Transplant..

[39] DiPaola F.W., Schumacher K.R., Goldberg C.S., Friedland-Little J., Parameswaran A., Dillman J.R. (2017). Effect of Fontan operation on liver stiffness in children with single ventricle physiology. Eur. Radiol..

[40] Guerrero-Chalela C.-E., Therrien J., Grossman Y., Guo L., Liu A., Marelli A. (2023). Severe Fontan-Associated Liver Disease and Its Association with Mortality. J. Am. Heart Assoc..

[41] Schleiger A., Salzmann M., Kramer P., Danne F., Schubert S., Bassir C., Müller T., Müller H.-P., Berger F., Ovroutski S. (2020). Severity of Fontan-Associated Liver Disease Correlates with Fontan Hemodynamics. Pediatr. Cardiol..

[42] Balcar L., Semmler G., Scheiner B., Stättermayer A.F., Ćosić S., Schwabl P., Kazem N., Mandorfer M., Hülsmann M., Zuckermann A. (2023). Clinical course of congestive hepatopathy pre/post heart transplantation. Wien. Klin. Wochenschr..

[43] Rodriguez D.S., Mao C., Mahle W.T., Kanter K.R., Alazraki A., Braithwaite K., Rytting H., Caltharp S., Magliocca J.F., Romero R. (2021). Pretransplantation and Post-Transplantation Liver Disease Assessment in Adolescents Undergoing Isolated Heart Transplantation for Fontan Failure. J. Pediatr..

[44] Ybarra A.M., Khanna G., Turmelle Y.P., Stoll J., Castleberry C.D., Scheel J., Ballweg J.A., Ameduri R., Kimberling M., Makil E. (2021). Heterogeneous outcomes of liver disease after heart transplantation for a failed Fontan procedure. Pediatr. Transplant..

[45] Lewis M.J., Reardon L.C., Aboulhosn J., Haeffele C., Chen S., Kim Y., Fuller S., Forbess L., Alshawabkeh L., Urey M.A. (2023). Clinical Outcomes of Adult Fontan-Associated Liver Disease and Combined Heart-Liver Transplantation. J. Am. Coll. Cardiol..

[46] Vaikunth S.S., Concepcion W., Daugherty T., Fowler M., Lutchman G., Maeda K., Rosenthal D.N., Teuteberg J., Woo Y.J., Lui G.K. (2019). Short-term outcomes of en bloc combined heart and liver transplantation in the failing Fontan. Clin. Transplant..

[47] Karnsakul W., Wasuwanich P., Ingviya T., Laengvejkal P., Vasilescu A., Imteyaz H., Scheimann A.O. (2020). Clinical usage of serum albumin to ascitic fluid albumin gradient and ascitic fluid total protein in pediatric ascites. Clin. Res. Hepatol. Gastroenterol..

